# Extended-spectrum beta-lactamase − producing enterobacteriaceae in the intensive care unit: acquisition does not mean cross-transmission

**DOI:** 10.1186/s12879-016-1489-z

**Published:** 2016-04-13

**Authors:** Mikael Alves, Astrid Lemire, Dominique Decré, Dimitri Margetis, Naïke Bigé, Claire Pichereau, Hafid Ait-Oufella, Jean-Luc Baudel, Georges Offenstadt, Bertrand Guidet, Frédéric Barbut, Eric Maury

**Affiliations:** Service de Réanimation Médicale, Hôpital Saint-Antoine, Assistance Publique–Hôpitaux de Paris, 184 rue du Faubourg Saint-Antoine, 75012 Paris, France; Service de Microbiologie, Hôpital Saint-Antoine, Assistance Publique–Hôpitaux de Paris, 184 rue du Faubourg Saint-Antoine, 75012 Paris, France; Université Pierre et Marie Curie-Paris 6, Paris, France; Inserm-UPMC UMR S 1136, Paris, France

**Keywords:** Cross Infection, Polymerase chain reaction, beta-lactamase, Enterobacteriaceae, Anti-Bacterial Agents, ESBL-E

## Abstract

**Background:**

In intensive care unit (ICU), infection and colonization by resistant Gram-negative bacteria increase costs, length of stay and mortality. Extended-spectrum beta-lactamase − producing Enterobacteriaceae (ESBL-E) is a group of pathogens increasingly encountered in ICU setting. Conditions that promote ESBL-E acquisition are not completely understood. The increasing incidence of infections related to ESBL-E and the unsolved issues related to ESBL-E cross-transmission, prompted us to assess the rates of referred and acquired cases of ESBL-E in ICU and to assess patient-to-patient cross-transmission of ESBL-E using a multimodal microbiological analysis.

**Methods:**

During a 5-month period, all patients admitted to a medical ICU were tested for ESBL-E carriage. A rectal swab was performed at admission and then twice a week until discharge or death. ESBL-E strains were analyzed according to antibiotic susceptibility pattern, rep-PCR (repetitive-element Polymerase chain reaction) chromosomal analysis, and plasmid PCR (Polymerase chain reaction) analysis of ESBL genes. Patient-to-patient transmission was deemed likely when 2 identical strains were found in 2 patients hospitalized simultaneously in the ICU.

**Results:**

Among the 309 patients assessed for ESBL-E carriage on admission, 25 were found to carry ESBL-E (importation rate: 8 %). During follow-up, acquisition was observed among 19 of them (acquisition rate: 6.5 %). Using the multimodal microbiological approach, we found only one case of likely patient-to-patient ESBL-E transmission.

**Conclusions:**

In unselected ICU patients, we found rather low rates of ESBL-E referred and acquired cases. Only 5 % of acquisitions appeared to be related to patient-to-patient transmission. These data highlight the importance of jointly analyzing phenotypic profile and molecular data to discriminate strains of ESBL-E.

**Electronic supplementary material:**

The online version of this article (doi:10.1186/s12879-016-1489-z) contains supplementary material, which is available to authorized users.

## Background

In intensive care unit (ICU), Gram-negative bacterial resistance to antibiotic therapy increases costs, length of stay and mortality [1, 2]. One major mechanism of resistance is related to extended-spectrum beta-lactamase (ESBL) production. Since the first report in mid-1980s, the incidence of ESBL-producing Enterobacteriaceae (ESBL-E) has been increasing worldwide [3]. “Old” ESBL (derived from SHV and TEM families) were, until the year 2000, essentially related to *Klebsiella pneumoniae* and *Escherichia coli*, which were responsible for nosocomial infections, mostly in the ICU setting [4, 5]. *Escherichia coli* thereafter became dominant among ESBL-E. Interestingly, the change of dominant species occurred concomitantly with the emergence of enzymes that belong to the CTX-M family. These “new” ESBL have superseded the TEM- and SHV-related enzymes, and their incidence is currently increasing in the community setting [6, 7].

ESBL-E community carriage and/or hospital acquisition rates vary worldwide. In Madagascar, more than 10 % of healthy volunteers carry an ESBL-E strain [8]. In Spain, ESBL-E carriage increases between 1991 and 2003 of 1–5 % among ambulatory patients and 1–12 % among hospitalized patients [9]. In France, carriage of ESBL-E is about 1 % in healthy volunteers [10] and up to 6 % in patients admitted to a medical ward [11].

Acquisition can be due to transmission from one patient to another via health care worker’s hands. This pattern is largely accepted for glycopeptide-resistant *Enterococcus* (GRE), and prevention programs designed to minimize cross-transmission, have reduced this mode of acquisition [12–15]. Programs designed to prevent the spread of “old” ESBLs are less convincing and even discordant with “new” ESBLs epidemiology [16, 17]. Other patterns of acquisition include antibiotic pressure [18], and the use of antibiotics in food animal breeding [19]. Regarding the environment, some authors report possible GRE and Methicilin Resistant *Staphylococcus aureus* (MRSA) contamination from bedroom furniture and medical devices [20, 21], which can be decreased by reinforced environmental cleaning [22].

The relative contributions of all these factors to ESBL-E acquisition are incompletely understood [23]. Contact isolation measures are usually applied to ESBL-E carriers [14] but are potentially harmful for patients and their effectiveness is even debated [24].

The increasing incidence of infections related to community-acquired or nosocomial ESBL-E and the issues raised by data on patient-to-patient transmission, prompted us to assess colonization and acquisition rates of ESBL-E and to characterize ESBL-E cross-transmission using microbiological multimodal analysis.

## Methods

### Study design and patient population

This study was approved by the Comité de Protection des Personnes de l’Hôpital Saint-Antoine.

We assessed in a multimodal analysis, microbiological samples collected during routine screening for multidrug-resistant bacteria in the medical ICU of a 660-bed tertiary teaching hospital, during a period of 5 consecutive months (March 15^th^ to August 15^th^, 2011). The medical ICU has 3 units containing each 6 single beds. Two physicians are in charge of a Unit. A nurse cares for 3 patients. All patients admitted to the medical ICU were given information on the study and their (or next of kin) oral consent was obtained. Every patient underwent rectal swab screening for ESBL-E carriage at admission and then twice a week until ICU discharge. Enhanced hygiene measures (protective gowns, gloves, ESBL-E announcing stickers) were applied in the case of patients colonized and/or infected by ESBL-E and preventively in patients considered at risk for ESBL-E carriage.

### Microbiological methods

Screening for ESBL-E was performed by inoculating rectal swabs on selective medium supplemented with ceftazidime (bioMérieux, Marcy l’Etoile, France). After 24 h at 37 °C, the species were identified by MALDI-TOF (*Matrix-Assisted Laser Desorption/Ionisation*, *time-of-flight mass spectrometry*) analysis. Antibiotic susceptibility was tested using the standard agar diffusion method on Mueller-Hinton agar (Bio-Rad, Marnes-la-Coquette, France) according to CA-SFM 2012 guidelines. The following antibiotics (bioRad) were tested: amoxicillin, amoxicillin-clavulanate, ticarcillin, piperacillin, piperacillin-tazobactam, cefalotine, cefoxitine, moxalactam, cefotaxime, ceftazidime, aztreonam, cefepime, imipenem, ertapenem, meropenem, gentamicin, tobramycin, netilmicin amikacin, nalidixic acid, ofloxacin, ciprofloxacin and sulfamethoxazole. The double-disk synergy method was used to confirm ESBL production [25]. All ESBL-E − producing isolates were stored at −80 °C.

### Molecular methods

Clonal relationships of ESBL-E isolates were investigated using the DiversiLab^®^ fingerprinting system (bioMérieux, Marcy l'Etoile, France), a commercially available repetitive-element (rep)-PCR tool [26] successfully used for the typing of ESLB-E. This technique is faster than and as discriminating as pulsed-field gel electrophoresis [27, 28]. After thawing, strains were isolated and incubated at 37 ° C for 24 h. DNA was extracted using an UltraClean DNA isolation kit^®^ (MO-BIO, USA). The DNA solutions were then normalized at a concentration of 35 ng/mL. For the study, we used specific rep-PCR kits respectively for *Escherichia coli*, *Enterobacter* ssp*.*, and *Klebsiella* spp*.* species. PCR cycling parameters for all the kits were similar: an initial denaturation at 94 °C for 2 min, 35 cycles of denaturation at 94 °C for 30 s, hybridization (at 55 °C for *Enterobacter* spp. and *Klebsiella* spp*.* and at 50 °C for *Escherichia coli*) for 30 s, extension at 70 °C for 90 s and a final extension at 70 °C for 3 min. DNA amplicons were separated by a bioanalyzer (Agilent Technologies, Les Ulis, France).

DiversiLab^®^ software was used for analysis of the results. This used the Pearson correlation coefficient to determine distance matrices and the UPGMA method (Unweighted Pair Group Method with Arithmetic Mean) to create dendrograms. Reports were automatically generated including dendrogram, electropherogram, virtual gel images and scatter plots to aid in data interpretation. A cluster was defined by a set of strains having both a similarity coefficient equal to or greater than 95 % and difference less than 2 peaks on the electropherogram.

In order to complement the results, all ESBL genes were characterized. DNA preparation and multiplex PCRs, previously developed to detect the most frequent widespread beta-lactamase genes encoding the ESBL (SHV-, TEM- and CTX-M-types), were performed as previously described [29]. PCR products were purified using the ExoSap purification kit (Illustra EXOSTAR-1 Step, Duscher, Brumath, France). All PCR products were subjected to bidirectional DNA sequencing using the BigDye terminator 3.1 cycle sequencing kit (Applied Biosystems, Foster City, CA, USA). Each sequence was aligned using Applied Biosystems SeqScape® software. The nucleotide sequences and deduced protein sequences were analyzed with the BLAST and FASTA programs of the National Centre for Biotechnology Information.

### Definitions

According to the results of culture and clinical data (date of admission and discharge, location and transfer within the unit, and previous admissions), each ESBL-producing strain was classified as referred or acquired in the unit. Referred cases were those that met any of the following criteria: patient with ESBL-producing strain found at admission; patient with ESBL-producing strain found from a clinical sample within 48 h following ICU admission; and/or history of colonization and/or infection with an ESBL-producing strain (defined by positive rectal swab and/or positive microbiologic exam for ESBL-E at a previous stay). The rate of referred cases was computed for patients who underwent sampling at admission. ESBL-E acquired cases were defined by the identification 72 h after admission of one ESBL-E strain among patients with negative admission screening. Identification of several novel ESBL-E strains with different beta-lactamases at the same sampling was considered as several acquisitions. Acquisition rate was computed for patients in whom admission sampling had been performed. ESBL-E acquired cases occurring among patients carrying referred strains was defined as identification 72 h after admission and during follow-up of an ESBL-E strain different from the one identified on admission sampling. Cross-transmission (patient-to-patient transmission) was recorded when strains isolated among 2 patients were similar in both beta-lactamase gene characterization and rep-PCR analysis. Moreover, the 2 patients ICU stays had to overlap.

### Statistics

Quantitative values were expressed as the median with the interquartile range [25–75 %]. Rates for referred cases and acquired cases were calculated considering only patients whose screening samples at admission were available. For identification of cross-transmission, all ESBL-E strains isolated during the study were considered and acquisitions were studied, one by one, on the basis of antibiotic susceptibility pattern, ESBL-type rep PCR profile, and the patient’s period of ICU stay. The Mann–Whitney test was used to compare quantitative values. Proportions were compared using the Chi-2 test and/or the Fisher exact test, as appropriate.

## Results

### Demographic data

During the 5-month study period, 432 patients were admitted to the medical ICU (age 64 years [51–76], male/female ratio 56 %, IGSII 38 [27–52]) (Table 1). They were mainly admitted for medical reasons (90 %): respiratory failure (54 %), septic shock (21 %), coma (14 %), postoperative major surgery (5 %), and others pathologies (6 %). Patients came from a medical ward (54 %), from surgery (5 %), and from home (41 %). Length of stay was 5 days [3–7], and mortality was 18 %.

**Table 1:** Demographic data

	Patients without positive ESBL-E sample	ESBL-E referred patients	ESBL-E acquired patients	*p*
Number	268	25	19	
Age (years)	63 [51–75]	65 [59–76]	67 [49–78]	0.39 ^a^ 0.52 ^b^
Sex ratio (M/F)	57	48	68	0.40 ^a^ 0.35 ^b^
IGS 2	38 [27–51]	42 [33–47]	42 [36–51]	0.55 ^a^ 0.39 ^b^
ICU Mortality (%)	19	16	15.7	1 ^a^ 1 ^b^
Length of stay (days)	4 [3–6]	7 [6–9]	12 [8–23]	<0.005^a^ <0.005 ^b^

(Age, IGS 2 and length of stay are expressed as medians, ^a^comparison between ESBL-E imported patients and patients without positive ESBL-E cultures, ^b^comparison between ESBL-E acquired patients and patients without positive ESBL-E cultures)

### ESBL-E referred cases/acquired cases (Fig. 1)

**Fig. 1:**
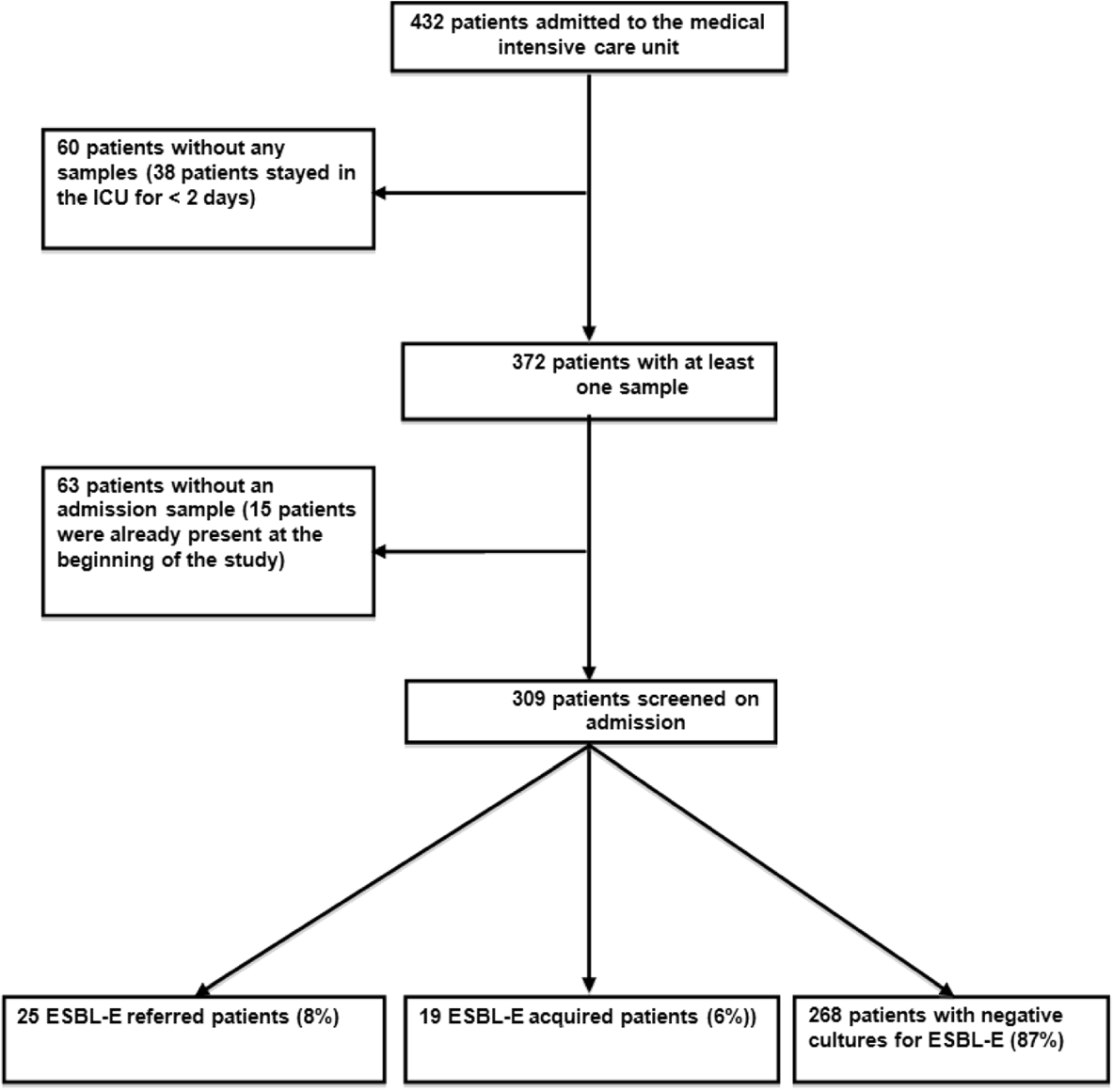
Flow chart

#### Patients

On admission, rectal swabs were performed in 309/432 patients (72 %). Twenty-five were positive for ESBL-E (referred cases rate 8 %) with 25 strains isolated from 25 patients. During follow-up of these 309 patients, 20 acquisitions were observed with 20 strains isolated from 19 patients (acquired cases: 6.5 %). Acquisition was observed in 3/19 of patients known to carry an ESBL-E upon admission. Acquisition occurred after 7 days [4–15] and ICU length of stay was significantly higher in patients who acquired ESBL-E (12 vs. 4 days, *p < 0.005*). Twelve patients had a history of colonization related in their records. For six of them, an ESBL-E was identified on a rectal swab during ICU stay. Among the 123 patients for whom admission samples were not available (and who therefore were not taken into account in computing rates of imported cases at admission and acquired cases), subsequent follow-up revealed carriage of ESBL-E strains in 10 of them.

### Strains

Fifty-two ESBL-producing strains of Enterobacteriaceae including *E. coli* (*n* = 27), *E. cloacae* (*n* = 11), K*. pneumoniae* (*n* = 10), and *K. oxytoca* (*n* = 4) were isolated during the study.

### Phenotypic analysis

Phenotypic classification of the strains was based on Antibiotic susceptibility profile defined as: I: gentamicin- and amikacin-sensitive; II: gentamicin-resistant and amikacin-sensitive; III: gentamicin-sensitive and amikacin-resistant; a: ciprofloxacin-sensitive; b: ciprofloxacin-resistant. This allowed discriminating 5 different antibiotic resistance patterns for *E. coli*, 4 for *E. cloacae*, 5 for *K. pneumoniae*, and 2 for *K. oxytoca* (Additional file 1).

### Molecular analysis

Molecular typing derived from rep-PCR analysis discriminated several clusters respectively among *E coli, E cloacae, K pneumoniae, K oxytoca* isolates.

**Fig. 2:**
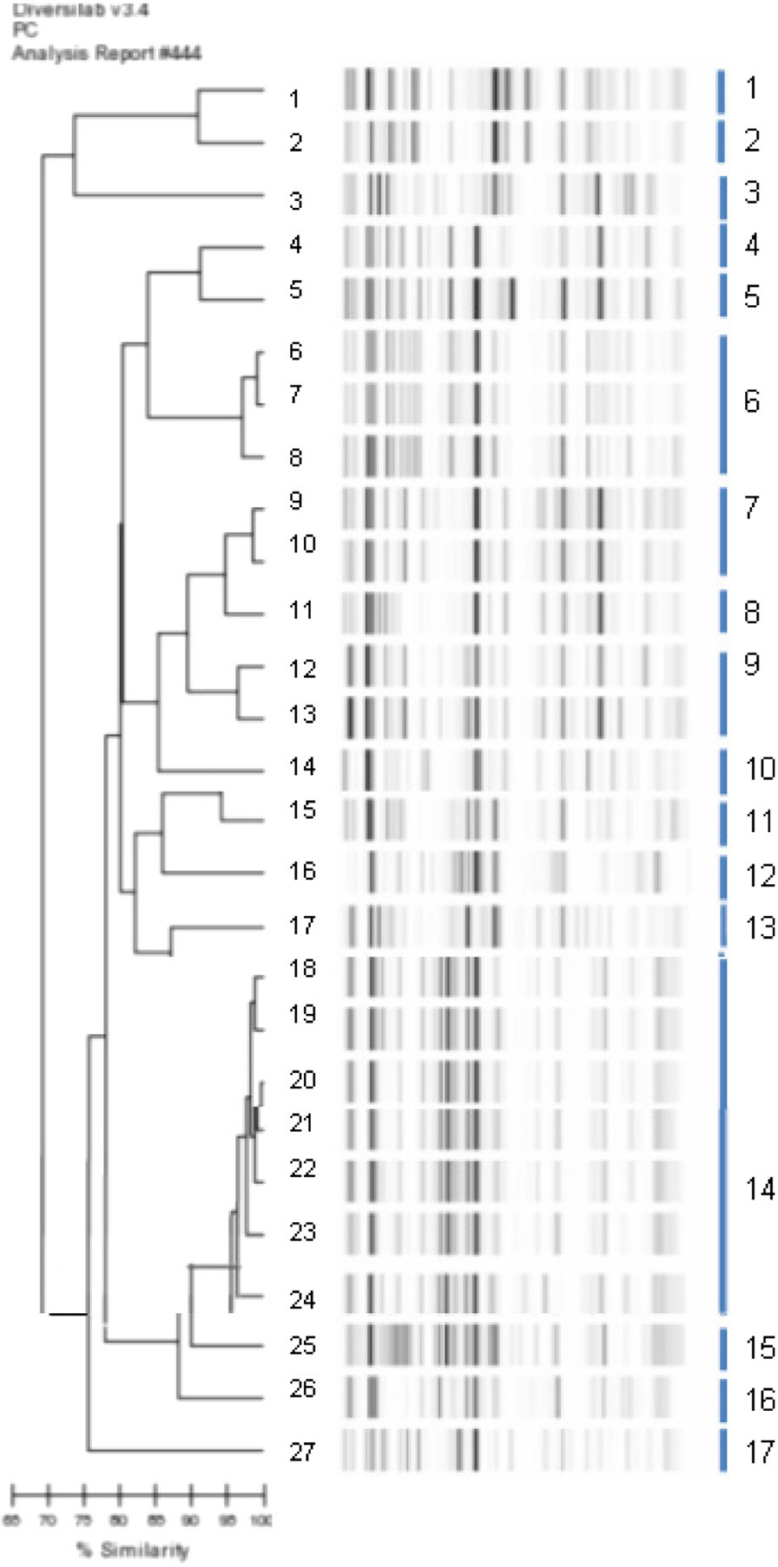
Dendrogram analysis and virtual gel images of DiversiLab rep-PCR fingerprinting system (bioMérieux) for the 27 *E. coli* isolates
- For *E. cloacae,* (Fig. 3); molecular typing discriminated 7 clusters: 5 clusters each containing only one isolate, one cluster with 2 isolates, and one cluster observed with 4 isolates.

- For the 27 *E. coli* isolates, (Fig. 2), 17 clusters were individualized: 13 of them with a single isolate, whereas clusters 6, 7, 9 and 14 contained respectively 3, 2, 2, and 7 isolates.

**Fig. 3:**
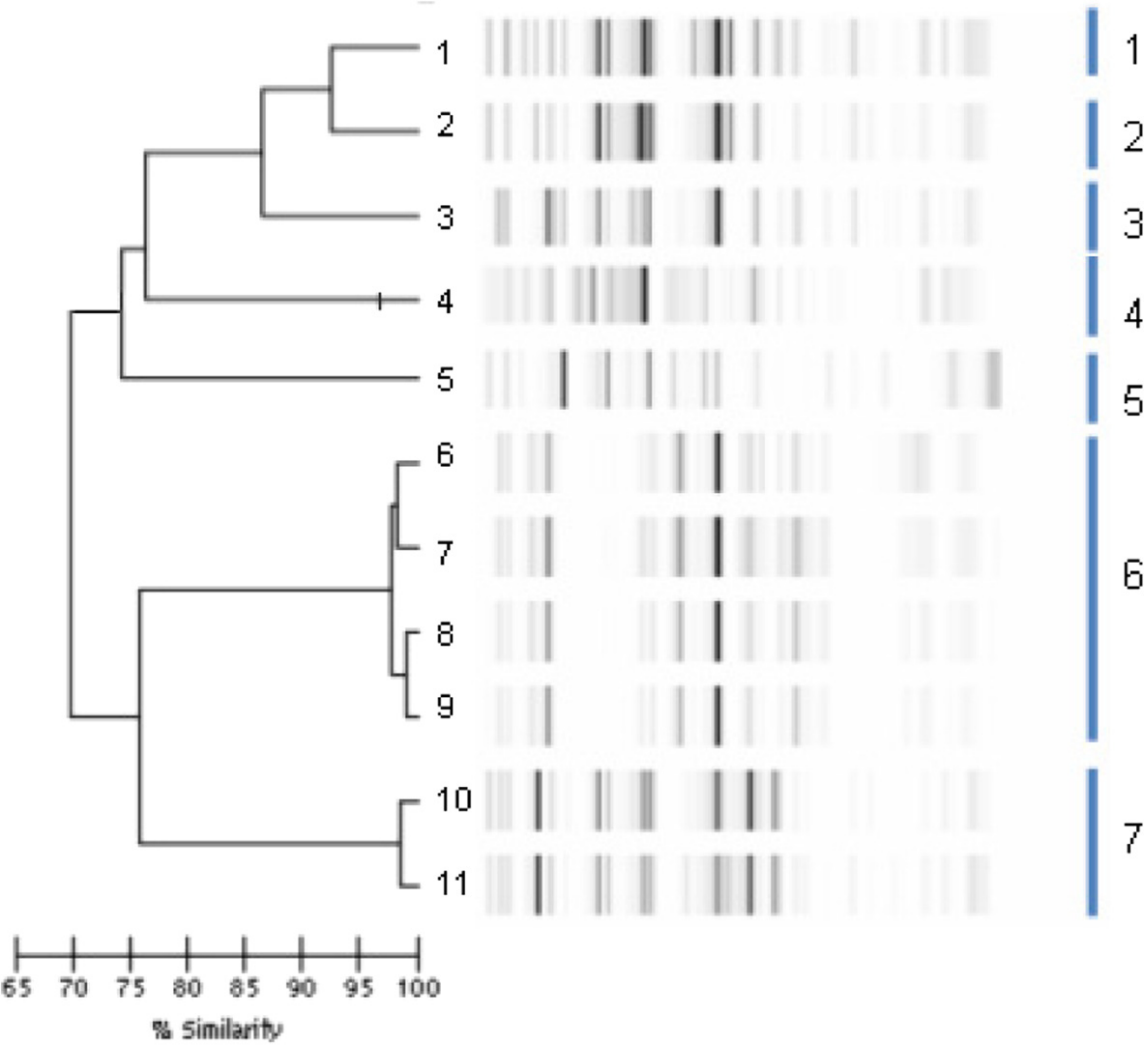
Dendrogram analysis and virtual gel images of DiversiLab rep-PCR fingerprinting system (bioMérieux) for the 11 *E. cloacae* isolates
- Three of the 10 *K. pneumoniae* isolates were part of the same cluster whereas each remaining seven were from a different cluster (Fig. 4).

**Fig. 4:**
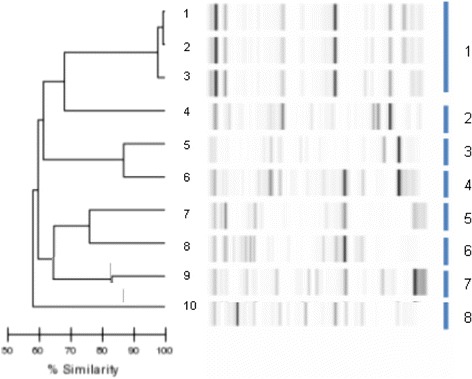
Dendrogram analysis and virtual gel images of DiversiLab rep-PCR fingerprinting system (bioMérieux) for the 10 *K. pneumoniae* isolates
- The 4 *K. oxytoca* isolates each showed a distinct cluster (Fig. 5).

**Fig. 5:**
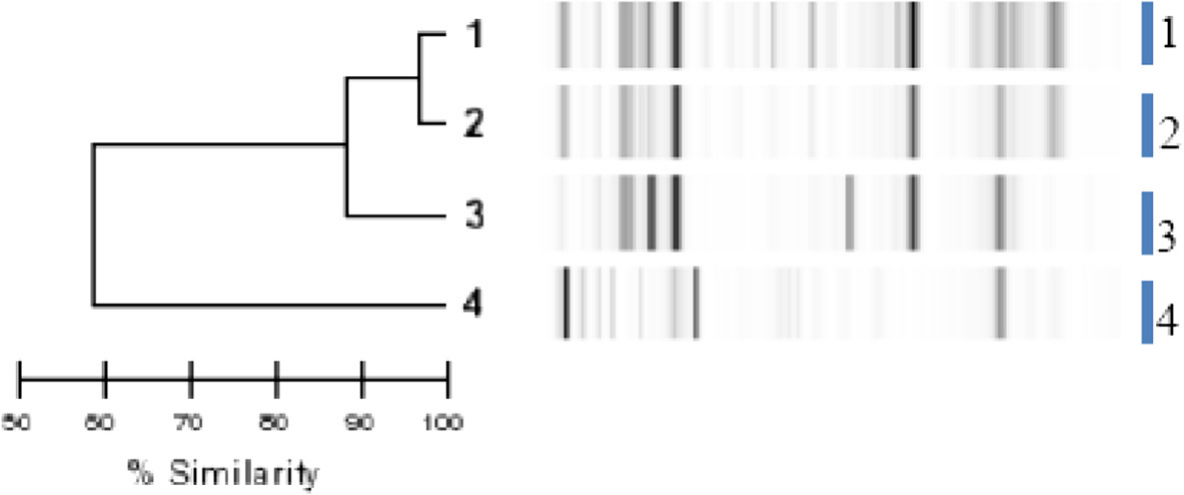
Dendrogram analysis and virtual gel images of DiversiLab rep-PCR fingerprinting system (bioMérieux) for the 4 *K. oxytoca* isolates

Our results showed CTX-M enzymes to be the most frequent ESBL-types (Additional file 1). ESBL for *E. coli* included 21 CTX-M (8 CTX-M-15, 7 CTX-M-1, 1 CTX-M-3, and 5 CTX-M-14), 2 TEM-type enzymes and 2 SHV-12. For *Klebsiella* species, 8/10 *K. pneumoniae* produced CTX-M-15 whereas the 4 *K. oxytoca* produced SHV-12. Finally, 10/11 *E. cloacae* were CTX-M-15 producers.

Integrating all molecular results has allowed showing that in the same cluster, analysis of the genes encoding ESBL resistance is required to differentiate strains there between. For example, on *E. coli*, cluster 14 contains 1 strain with resistance encoded by CTX-M-27, 5 strains with resistance encoded by CTX-M-15, and 1 strain with resistance encoded by CTX-M-14). Moreover, we observed strains of the same species with similar resistance profiles that did not derive from the same cluster (Additional file 1).

### Cross-transmission

Eleven ESBL-E acquisitions out of 19 (19 patients) corresponded to an ESBL-E that was found neither on hygiene sampling nor on clinical sampling performed during the study period, which makes patient-to-patient transmission very unlikely.

One patient acquired an *E. coli* strain, which belonged to a cluster, found on admission sampling in another patient admitted to our ICU 10 days after. Whereas they belonged to the same cluster, these 2 strains exhibited both a different pattern of antibiotic susceptibility to aminoglycosides and a different ESBL type.

An *E. cloacae* acquisition was observed in one patient, 13 days before the admission of another patient to the same unit from whom admission screening samples indicated the same strain (Cluster 7), making patient-to-patient cross-transmission very unlikely.

Four ESBL-Es were acquired during the study period (similar cluster and ESBL analysis) in the same unit (one *E. coli* (Cluster 14), 2 *E. cloacae* (Cluster 6), one *K. pneumoniae* (Cluster 1). However, there was no overlap in the ICU stays of these patients, suggesting that patient-to-patient transmission was very unlikely.

*E. coli* (E18) was found in follow-up sampling in one patient (acquisition). This cluster (14) was found in samples from another (E22) patient. However, molecular analysis individualized 2 different ESBL-types (CTX-M-27 and CTX-M-15).

Finally, according to molecular (rep PCR and ESBL PCR) and phenotypic typing and bearing in mind geographic and temporal compatibility, only one case of highly probable patient-to-patient transmission occurred during the study (*K. pneumoniae*, Cluster 1).

## Discussion

In this study, we assessed the rates of referred ESBL-E at admission and ESBL-E acquisition during ICU stay. Our study complements the few ones that have investigated *Enterobacteriaceae* acquisition and cross-transmission using a multimodal approach [16, 17, 30].

In our study, ESBL-E carriage at admission and acquisition during ICU stay were rare, occurring in 8 % and 6 % of patients, respectively. Using phenotypic and molecular typing, and considering geographic and temporal compatibility, we observed only one possible case of patient-to-patient transmission among the 19 patients who acquired an ESBL-E during their ICU stay.

ESBL-E imported cases accounted for less than 10 % in the ICU. This rate is in keeping with data published during the last decade. In 2007, Buke et al. reported at admission ESBL-E carriage in less than 8 % of patients who stayed for more than a month in a French hospital [31]. In Spain, carriage of ESBL-E among hospitalized patients was 8.2 % in 2010 [32]. The acquisition rate was 6 % in the present study. Despite recently observed emergence of community-acquired ESBL-E, in-hospital ESBL-E acquisition remained stable over years. This could suggest that either hygiene measures are effective or that the role attributed to patient-to-patient transmission of ESBL-E in the acquisition pattern is weak [3]. In the present study, using a multimodal microbiological approach, the observed acquisition rate was very low. Indeed, among 309 patients screened on admission to the ICU, cross-transmission was considered likely in only one patient. This result is certainly due to many issues. First, the present study was conducted during a period of very low endemicity. In consequence, we cannot exclude the possibility that different results may be observed during a period of high endemicity or during an outbreak. In addition, isolation measures applied in a preventive manner could have modified the cross-transmission rate [33].

A recently published study using a similar methodology reported possible patient-to-patient transmission for only 3 patients in 69 cases of ESBL-E acquisition [34]. In an older French study, cross-transmission rate was higher with over 85 % of strains acquired, but this study was only based on antibiotic susceptibility [35].

We compared both phenotypic analysis, based on antibiotic susceptibility, and molecular analysis. We confirmed that the capacity of antibiotic susceptibility analysis to discriminate similar strains is weak. Moreover, we confirm that strain with similar antibiotic susceptibility patterns and which belong to the same cluster (relying on rep-PCR) can differ only by their ESBL production. This is the case for 2 pairs of *E. coli* isolates (18 and 22) and (12 and 13) that carried (CTX-M 27 and CTX-M 15) and (SHV 12 and CTX-M 14), respectively. These observations are consistent with the fact that ESBL genetic support is mainly extrachromosomal (plasmid-related). As reported by several other authors, we observed this phenomenon only for *E. coli* strains [16, 17]. Our data highlight the importance-combined analysis of phenotypic profile and molecular data to discriminate ESBL-E strains.

The present study, however, has several limitations. First, despite its worldwide use, the rectal swab is of limited sensitivity in detecting ESBL-E carriage. This issue is well documented for vancomycin-resistant enterococci (VRE), for which probability of detection is inversely proportional to the number of colony-forming units in rectal swabs [36]. The risk of false-negative results with rectal swabs is probably lower for ESBL-E due to the virulence and larger inocula observed with these bacteria [23]. However, Harris et al. reported a non-detection rate of 69 % for *E. coli* and *Klebsiella spp.* [37]. In the present study, when rectal swabs were considered by laboratory technicians as containing insufficient amount of stool, they were discarded and rectal sampling was immediately re performed.

Antimicrobial exposure could have contributed to change duration of carriage and limited the detection during the study. Among patients with at least one positive rectal swab, 48 % had successively several negative rectal swabs. For 66 % of them, active antibiotics on isolated ESBL-E were administered when negative screening samples were observed. Duration of colonization by the ESBL-E strain is difficult to assess. One study showed that the median duration of carriage of ESBL-producing *Klebsiella pneumoniae* was 160 days after hospital discharge [38]. This study showed a significant variability between patients. Haverkate et al. showed that the median duration of carriage of highly resistant ESBL Enterobacteriaceae was 1.4 months in patients colonised with a MDRO during a previous stay in the ICU [39]. Currently, there is no formal data to recommend routine screening to assess the persistence of carriage.

Second, potential increased risk of acquisition, transmission and carriage which antimicrobial may contribute exposure was not looked for.

Third, sampling at admission was analysable in only 70 % of patients. The pertinence of the carriage rate we observed would have been probably greater with a higher percentage of patients sampled on admission. However, we included 86 % of all patients in the cross-transmission analysis, a percentage similar to that in most studies designed to characterize multiresistant strains cross-transmission.

Median length of stay was short (4 days), which is usual length of stay of patients admitted to our unit. However, we observed that patients who acquired ESBL-E had a significantly longer ICU stay (12 days). A similar study in patients who spend longer in the ICU may, therefore, indicate a different acquisition rate. Furthermore, criteria as those used to define cross-transmission can be appropriate for an ICU with short median lengths of stay but are not necessarily applicable in other ICU settings.

Finally, it should be highlighted that patient-to-patient transmission was low in our study. This can be related to several issues. Protective isolation measures were applied as soon as ESBL-E detection and immediately at admission in patients at high risk of carrying ESBL-E. In several cases of acquisition, patient-to-patient transmission initially deeming likely (because the strains were identical) was finally ruled out because there was no overlap of patient ICU stays. This type of acquisition could account for a possible acquisition from inanimate surfaces since ESBL-E can survive in such conditions for a long time [21].

## Conclusions

In this observational study conducted in patients with a relatively short length of stay in the ICU, low rates of both ESBL-E carriage at admission and ESBL-E acquisition during ICU stay were observed. Using a multimodal microbiological approach, we identified only one possible case of patient-to-patient transmission of ESBL-E among the 19 acquisitions observed during ICU stay. These data highlight the importance of jointly analyzing phenotypic and molecular profiles, to discriminate strains of ESBL-E before assuming that they are identical.

## Additional file

Additional file 1: Characterization of ESBL-E. (DOCX 92 kb)

## Abbreviations

CA-SFM: comite de l’antibiogramme – societe française de microbiologie
DNA: deoxyribonucleic acid
ESBL-E: extended-spectrum beta-lactamase − producing Enterobacteriaceae
ESBL: extended-spectrum beta-lactamase
ICU: intensive care unit
IGSII: index gravity score
IRB: institutional review board
GRE: glycopeptide-resistant *Enterococcus*
MALDI-TOF: matrix-assisted laser desorption/ionisation, time-of-flight mass spectrometry
MRSA: methicillin resistant *Staphylococcus aureus*
PCR: polymerase chain reaction
Rep-PCR: repetitive-element PCR
UPGMA: unweighted pair group method with arithmetic mean
