# Supplementary material for: Extended-spectrum beta-lactamase − producing enterobacteriaceae in the intensive care unit: acquisition does not mean cross-transmission
Source: BMC Infect Dis. 2016 Apr 13;16:147. doi: 10.1186/s12879-016-1489-z (PMC4831109; doi:10.1186/s12879-016-1489-z)
Supplement: Additional file 1: — Characterization of ESBL-E. (DOCX 92 kb) [file 12879_2016_1489_MOESM1_ESM.doc]

**Additional file 1: Characterization of ESBL-E**

Antibiotic susceptibility profile is defined as, I: gentamicin- and amikacin-sensitive; II: gentamicin-resistant and amikacin-sensitive; III: gentamicin-sensitive and amikacin-resistant; a: ciprofloxacin-sensitive; b: ciprofloxacin-resistant

| *Escherichia coli* strain number^1^ | | Antibiotic susceptibility profile | Cluster | ESBL type |
| --- | --- | --- | --- | --- |
|  | E1 | Ib | 1 | CTX-M-27 |
|  | E 2 | Ib | 2 | CTX-M-15 |
|  | E 3 | Ia | 3 | CTX-M-15 |
|  | E 4 | IIa | 4 | TEM-15 |
|  | E 5 | Ia | 5 | CTX-M-1 |
|  | E 6 | Ia | 6 | CTX-M-1 |
|  | E 7 | Ia | 6 | CTX-M-1 |
|  | E 8 | Ia | 6 | CTX-M-1 |
|  | E 9 | Ib | 7 | CTX-M-3 |
|  | E 10 | IIa | 7 | CTX-M-14 |
|  | E 11 | Ia | 8 | SHV-12 |
|  | E 12 | Ia | 9 | SHV-12 |
|  | E 13 | Ia | 9 | CTX-M-14 |
|  | E 14 | Ib | 10 | CTX-M-14 |
|  | E 15 | IIb | 11 | CTX-M-15 |
|  | E 16 | IIa | 12 | TEM-15 |
|  | E 17 | Ia | 13 | CTX-M-1 |
|  | E 18 | Ib | 14 | CTX-M-27 |
|  | E 19 | IIIb | 14 | CTX-M-15 |
|  | E 20 | IIIb | 14 | CTX-M-15 |
|  | E 21 | IIIb | 14 | CTX-M-15 |
|  | E 22 | Ib | 14 | CTX-M-15 |
|  | E 23 | IIa | 14 | CTX-M-14 |
|  | E 24 | IIIb | 14 | CTX-M-15 |
|  | E 25 | Ib | 15 | CTX-M-14 |
|  | E 26 | Ia | 16 | CTX-M-1 |
|  | E 27 | Ia | 17 | CTX-M-1 |
| *E. cloacae*  Strain number^2^ | |  |  |  |
|  | EC1 | IIa | 1 | CTX-M-15 |
|  | EC 2 | IIb | 2 | CTX-M-15 |
|  | EC 3 | IIb | 3 | CTX-M-15 |
|  | EC 4 | IIb | 4 | CTX-M-15 |
|  | EC 5 | IIIa | 5 | TEM-3 |
|  | EC 6 | IIb | 6 | CTX-M-15 |
|  | EC 7 | IIb | 6 | CTX-M-15 |
|  | EC 8 | IIb | 6 | CTX-M-15 |
|  | EC 9 | IIb | 6 | CTX-M-15 |
|  | EC 10 | IIb | 7 | CTX-M-15 |
|  | EC 11 | IIb | 7 | CTX-M-15 |

| *K. pneumonia*  Strain number^3^ | | Antibiotic susceptibility profile | Cluster | β-Lactamase |
| --- | --- | --- | --- | --- |
|  | KP1 | IIIb | 1 | CTX-M-15 |
|  | KP 2 | IIIb | 1 | CTX-M-15 |
|  | KP 3 | IIIb | 1 | CTX-M-15 |
|  | KP 4 | IIb | 2 | CTX-M-15 |
|  | KP 5 | IIb | 3 | CTX-M-15 |
|  | KP 6 | IIa | 4 | TEM-15 |
|  | KP 7 | IIa | 5 | TEM-15 |
|  | KP 8 | IIb | 6 | CTX-M-15 |
|  | KP 9 | IIb | 7 | CTX-M-15 |
|  | KP 10 | IIIb | 8 | CTX-M-15 |
| *K. oxytoca* strain number^4^ | |  |  |  |
|  | KO1 | IIa | 1 | SHV-12 |
|  | KO 2 | IIIa | 2 | SHV-12 |
|  | KO 3 | IIa | 3 | SHV-12 |
|  | KO 4 | IIa | 4 | SHV-12 |
|  |  |  |  |  |

(All strains were astreonam and cefotaxime-resistant, and imipenem-sensitive; ^1^ 18/27 piperacillin-tazobactam-sensitive, 22/27 gentamycin-sensitive, 23/27 amikacin-sensitive and 15/27 ciprofloxacin-sensitive; ^2^ piperacillin-tazobactam-resistant, 1 gentamycin-sensitive, 10 amikacin-sensitive and 2 ciprofloxacin-sensitive; ^3^ piperacillin-tazobactam-resistant, 5 gentamycin-sensitive, 6 amikacin-sensitive and 2 ciprofloxacin-sensitive; ^4^ piperacillin-tazobactam-resistant and ciprofloxacin-sensitive, 1 gentamycin-sensitive, and 3 amikacin-sensitive.)
